# Challenges in Using Ionic Liquids for Cellulosic Ethanol Production

**DOI:** 10.3390/molecules28041620

**Published:** 2023-02-08

**Authors:** Francieli Colussi, Héctor Rodríguez, Michele Michelin, José A. Teixeira

**Affiliations:** 1 CEB—Centre of Biological Engineering, University of Minho, 4710-057 Braga, Portugal; 2 LABBELS—Associate Laboratory, 4710-057 Braga, Portugal; 3 CRETUS, Department of Chemical Engineering, Universidade de Santiago de Compostela, E-15782 Santiago de Compostela, Spain

**Keywords:** pretreatment, one-pot process, lignocellulosic biomass, saccharification, biofuel

## Abstract

The growing need to expand the use of renewable energy sources in a sustainable manner, providing greater energy supply security and reducing the environmental impacts associated with fossil fuels, finds in the agricultural by-product bioethanol an economically viable alternative with significant expansion potential. In this regard, a dramatic boost in the efficiency of processes already in place is required, reducing costs, industrial waste, and our carbon footprint. Biofuels are one of the most promising alternatives to massively produce energy sustainably in a short-term period. Lignocellulosic biomass (LCB) is highly recalcitrant, and an effective pretreatment strategy should also minimize carbohydrate degradation by diminishing enzyme inhibitors and other products that are toxic to fermenting microorganisms. Ionic liquids (ILs) have been playing an important role in achieving cleaner processes as a result of their excellent physicochemical properties and outstanding performance in the dissolution and fractionation of lignocellulose. This review provides an analysis of recent advances in the production process of biofuels from LCB using ILs as pretreatment and highlighting techniques for optimizing and reducing process costs that should help to develop robust LCB conversion processes.

## 1. Introduction

The utilization of fuels is ubiquitous in many aspects of our current world, for example, in industry and transportation, just to mention two sectors in which it is critical. The replacement of fuels derived from fossil sources with fuels derived from renewable sources (biofuels) has evidently increased [1], but further advancement in the degree of this replacement is necessary to make adequate progress toward better fulfillment of the sustainable development goals set out in the 2030 Agenda of the United Nations [1].

The most archetypal biofuel is bioethanol, which can be obtained by the alcoholic fermentation of sugars, and these, at the same time, can be obtained by hydrolyzing polysaccharide-containing materials. In the first instance, industrial production from raw materials such as corn and sugarcane were considered, but unfortunate competition with the food market led to a switch toward non-edible raw materials. In this context, interest arose in ethanol production from lignocellulosic sources, known as cellulosic ethanol or second-generation (2G) ethanol. Lignocellulosic biomass (LCB) is basically an arrangement of three biopolymers: cellulose (35–50%), hemicellulose (20–35%), and lignin (10–25%), with the remaining fraction including proteins, oils, and ash [2]. LCB possesses a series of characteristics that make it an attractive feedstock for the industrial production of biofuels. Among others, it is well geo-distributed (especially if compared with key fossil sources such as petroleum) and widely available (as an agricultural or forestry by-product, as the direct product of sustainably managed forest lands, as the harvest of dedicated energy crop plantations, etc.), and nature generates it renewably at a much faster pace than the rate of consumption of fuels in human-related activities. 

However, one of the fundamental barriers to take advantage of LCB in the production of bioethanol or other biofuels is its recalcitrant character. The nature of the 3D matrix in which its three major biopolymeric constituents—cellulose, hemicellulose, and lignin—are interlinked hampers the accessibility of hydrolytic enzymes to the polysaccharides to generate the sugars for subsequent fermentation [3]. Thus, a pretreatment stage becomes necessary to improve such accessibility [4,5,6].

Multiple methods for the pretreatment of lignocelluloses, with the production of either biofuels or other chemicals of renewable origin as the ultimate goal, have been developed [5,7]. However, they typically involve the use of aggressive solvents, high temperatures and/or pressures, etc. Therefore, there is room for improvement of the sustainability credentials of the pretreatment stage through the development of cleaner methods with lower human and environmental impacts and better economic performance, offering good competitiveness [8,9,10,11]. In this regard, the emergence of active and multidisciplinary research on ionic liquids (ILs) and their appealing properties over the last couple of decades [12] has stimulated the envisioning of alternative solvent-based pretreatments [13], as is discussed in the present review.

## 2. Ionic Liquids for Better Methods for the Pretreatment of Lignocelluloses

**Figure 1:**
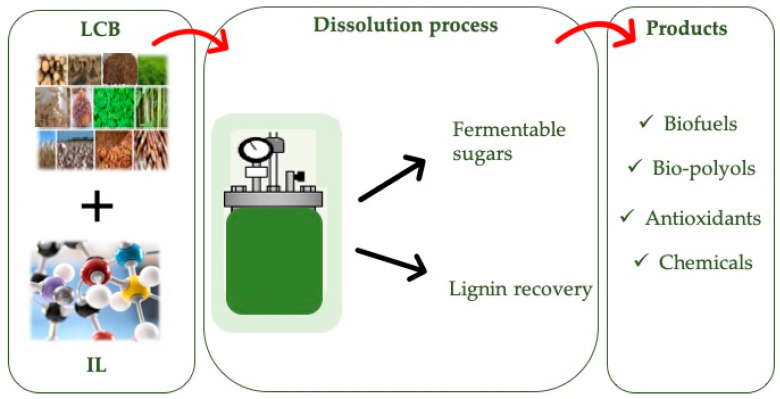
Application of ionic liquids (ILs) to the deconstruction and fractionation of lignocellulosic biomass (LCB).

The existing pretreatments of LCB present specific advantages but also a number of challenges that must be addressed. Among the different strategies, the chemical approach, such as the use of mineral acids and alkalis under harsh conditions, exhibits great potential to alter the lignocellulose structure [5,13]. ILs have the potential to become the basis of new chemical methods for the pretreatment of lignocelluloses [14]. These are salts with low melting temperatures (a mark of 100 °C is often considered, although many are liquid at room temperature and even far below) as a result of the nature of their constitutive cation–anion pairs, which largely frustrate the establishment of a high-energy crystalline network. Although it is difficult to generalize any single property to the entire family of ILs beyond the brief definition stated above, many of them typically possess a set of characteristics that make them attractive for use as neoteric solvents in a wide variety of processes. Examples of this are a practically negligible vapor pressure (thus avoiding the possibility of their loss by evaporation as well as the risk of generating dangerous atmospheres), reasonably good thermal stability, and quite broad temperature ranges in which they are stable liquids. Moreover, their properties (including physical, chemical, and biological properties) can be tuned to a reasonable extent by judicious choice of the constitutive ions, which has led to their coinage as “designer solvents” [14,15,16]. When LCB is pretreated, its components can be isolated and transformed into a variety of added-value products [17], as illustrated in Figure 1.

The consideration of ILs as potential alternative solvents for the pretreatment of lignocelluloses can be arguably traced back to work by Swatloski and collaborators, where some ILs had the capacity to dissolve cellulose in relevant amounts and under mild conditions [18]. Shortly after, this capacity was extended to the dissolution of lignocellulosic sources [8], including woody LCB [19,20], with even the possibility of achieving a certain degree of fractionation of their major biopolymers through appropriate sequential precipitation schemes [21]. In the meantime, ILs with the ability to selectively dissolve cellulose were also discovered [6,13,22,23]. 

Interestingly, the potential use of ILs in lignocellulose pretreatment processes does not have to be restricted to ILs capable of completely or selectively dissolving the main biopolymeric components under specific conditions. It has been recently reported that other ILs without this ability can also interact effectively with the constituting biopolymers, reducing, for instance, the crystallinity of cellulose or lignocellulosic materials [24,25]. This degree of crystallinity, along with other characteristics such as the degree of polymerization or the lignin distribution in the pretreated lignocellulosic material, is key for determining the accessibility of the polysaccharides to the enzymes in the process of enzymatic hydrolysis [26,27]. If the IL pretreatment is sufficiently effective, this type of hydrolysis will be preferred, in terms of the sustainability of the process.

For all of the above mentioned reasons, ILs have a solid possibility of contributing to the development of more sustainable LCB pretreatment methods in the production of biofuels, in particular cellulosic bioethanol, from LCB sources.

## 3. Saccharification of Lignocellulosic Biomass Pretreated with Ionic Liquids

The combination of IL and enzyme has impressive biotechnological and industrial potential. A set of these pairs is currently explored in order to gain an adequate understanding of the protein stability in the presence of ILs. 

The ILs are a potential compound in the dissolution of the LCB sources, and the yield of sugars increases considerably as the recalcitrance of the LCB decreases [27]. However, traces of the solvents that remain in the LCB after pretreatment can inhibit the enzymes by affecting the structure of proteins, their enantioselectivity, and consequently stability, preventing the effectiveness of enzymatic hydrolysis after pretreatment [6]. According to the literature, different types of LCB have already been tested with different types of available ILs, as shown in Table 1.

An and collaborators performed experiments with cholinium-based ILs in the following LCBs: rice straw, sugarcane bagasse, eucalyptus, pine, wheat straw, and corncob. Good results were verified for the tested lignocelluloses, resulting in significant improvements in the glucose yields (58–75%), but it was inefficient for the biodegradation of pine. In the rice straw treatment, 46% of the lignin was fractionated as lignin-rich material after pretreatment using cholinium argininate ([Ch][Arg]). This IL showed excellent recyclability, and the total recovery was as high as 75% after reuse for eight cycles. Besides, rice straw pretreated with the recycled IL remained highly digestible, and good glucose yields (63–75%) were achieved after its enzymatic hydrolysis [38]. Kassaye et al. [39] compared the alkaline and acidic hydrolysis of bamboo LCB pretreated with 1-butyl-3-methylimidazolium chloride ([Bmim][Cl]). They report that lignin recovery improved with the concentration of sodium hydroxide while LCB recovery got reduced owing to the partial loss of lignin and hemicellulose. Moreover, [Bmim][Cl] reduced the recalcitrance of bamboo, making it more susceptible to further acidic hydrolysis. The yield of total reducing sugars in untreated bamboo was 30%; alkaline treatment alone increased it to 64%; and the pretreatment with IL increased the yield of reducing sugars to 80% [38]. Nargotra and collaborators [40] reported a pretreatment of sunflower stalk LCB in a combinatorial regime involving alkali (NaOH) and [Bmim][Cl]. The result of this combination was approximately 60% higher when compared with separated treatments. This research group also performed biophysical studies of the LCB and showed huge differences between treatments [39]. Da Costa Lopes et al. optimized the process conditions for wheat straw pretreatment and hydrolysis of hemicellulose using a mixture of 1-ethyl-3-methylimidazolium hydrogensulfate ([Emim][HSO_4_]) and water [41]. At the optimized process conditions, a maximum yield of 80.5% pentoses (xylose and arabinose) was obtained in that study. The hydrolysis of hemicellulose was also studied by Carvalho et al. for the production of xylose and the conversion of xylose to furfural using [Bmim][HSO_4_]. The effect of reaction temperature was more profound on both xylose and furfural productions when compared with the effect of pretreatment time [42].

Hu et al. studied corn stalks incubated in a 50:50 mixture of 1-butyl-3-methylimidazolium tetrafluoroborate ([Bmim][BF_4_]) and water at 150 °C for 5 h and showed that the enzymatic hydrolysis efficiency increased up to 81.68%. Additionally, the removal of hemicellulose significantly destroyed the lignin–polysaccharide interactions, which was confirmed by FTIR and ^13^C NMR spectrograms [29]. Hashmi et al. compared the efficiency of autohydrolysis and the IL 1-butyl-3-methylimidazolium acetate ([Bmim][OAc]) as pretreatments for sugarcane bagasse in terms of delignification, cellulose crystallinity, and enzymatic digestibility. Glucan and xylan digestibility were determined to be 97.4% and 98.6% in [Bmim][OAc] (110 °C for 30 min) pretreated bagasse and 62.1% and 57.5% in bagasse autohydrolyzed at 205 °C for 6 min, respectively [30]. Other research showed that using [Emim][OAc] (120 °C for 30 min) followed by hydrolysis with commercial enzymes achieved higher glucan digestibility (87.0% and 64.3%) than untreated (5.5% and 2.8%) or water-treated (4.0% and 2.1%) energy cane bagasse to cellulose and hemicellulose, respectively. Biophysical methods were used to investigate the delignification and recalcitrance reduction of energy cane bagasse [36]. Rigual and co-workers studied the combination of autohydrolysis (150 °C, 175 °C, and 200 °C) and IL microwave (80 °C and 120 °C) treatments of eucalyptus wood. The ILs used here were [Emim][OAc], and the best condition was autohydrolysis at 175 °C and low IL at 80 °C, reaching a glucan digestibility of 84.4% [43].

## 4. One-Pot Integration of the Saccharification and Fermentation Stages

**Figure 2:**
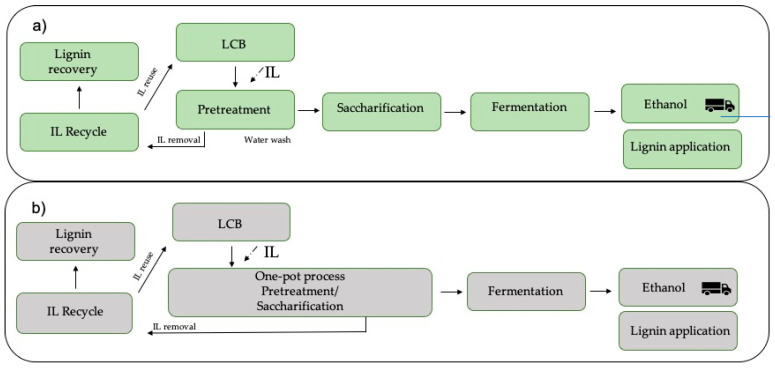
Block diagram for the production of bioethanol from lignocellulosic material. (**a**) Conventional pretreatment and saccharification in separated steps; (**b**) one-pot integrated IL process for biofuel production.

Pretreatments of LCB for sugar release are extensively studied. The target now is to obtain a more sustainable and economically viable system. The one-pot process (OPP) using ILs brings this proposal, integrating pretreatment and saccharification, followed by fermentation by the direct extraction of sugar and recovery of lignin as a by-product of the process [44,45], excluding the separations of the liquid–solids and washing phases after the pretreatment, reducing capital costs [46], and eliminating sugar losses during these separations (Figure 2).

Before realizing an affordable and scalable IL-based biomass conversion technology, issues such as IL toxicity, pH compatibility, and IL cost must be addressed. Most ILs that are effective biomass solvents are toxic to enzymes and microorganisms that are used in the downstream stages [47]. It is noteworthy that enzymatic inhibition by residual amounts of ILs in the OPP can occur. The toxicity of ILs is mainly dependent on the nature of the cation and its structural properties. Studies show that the alkyl chain in ILs increases its toxicity in most ecosystems. Some authors report that this toxic effect may be different in each organism, such as cell membrane rupture or even photosynthesis inhibition in plants [47,48] because ILs can act like an antibiotic or detergent and increase the osmotic pressure in microorganisms. This enzymatic inhibition might decrease the rate of saccharification and microbial fermentation. Second- and third-generation ILs (e.g., cholinium salts) are more sustainable and cause less toxicity, maintaining the integrity of the enzymes [8,48,49,50]. The pursuit of IL bio-derived and enzyme-tolerant cocktails is increasing and yielding excellent results [44]; then, the saccharification and fermentation can be combined, eliminating the separation of hydrolysates prior to the fermentation [47,51]. IL-tolerant engineered cellulases, cellulase-friendly ILs, or strategies for enzyme activation have been proposed [51]. Some microorganisms are showing good results in the presence of ILs, such as *Escherichia coli* [52] *Rhodosporidium toruloides* with the ability to metabolise a wide range of sugars and lignin-derived aromatic compounds [45,53]. Rigual and collaborators showed biocompatibility tests with ILs in *S. cerevisiae* and *R. toruloides*. The protic ILs are less toxic to yeasts when compared with cholinium lysinate ([Ch][Lys]) and are also more efficient for the treatment of eucalyptus than for pine, reaching up to 75% of digestibility [53,54].

Sundstrom et al. demonstrated an efficient OPP in a bench-scale and pilot bioreactor that keeps glucose and xylose yields around 80% and 60%, respectively. *R. toruloides* was chosen for the fermentation step due to its biological flexibility and IL tolerance, and also to the fact that it avoids utilization of extra water in the separation of phases, thus becoming a good alternative to OPP. It was also shown that this organism was able to consume glucose, xylose, and lactic acid in the presence of [Ch][Lys] [46]. 

Das [55] tested the use of seawater with [Ch][Lys] to convert sorghum into prespatane, an aviation biofuel, and compared it to the same treatment with freshwater. The best results were achieved with 10 wt% IL (88.5/86.9% glucose and 67.1/65.4% xylose in sea/freshwater). This is an excellent result because, in addition to increasing the sugar conversion, there is still no competition with water for human consumption [48]. Naz’s group analyzed the conversion of LCB into reducing sugars by OPP, using wheat straw as substrate and a pyridinium-based IL–metal salt system, reaching 70% of conversion to total reducing sugars (TRS) and 67 wt% of lignin removal in 2 h and 100 °C with 1-butyl-3-methylpyridinium chloride ([BMPy][Cl]) [56]. An OPP ethanolamine acetate pretreatment (HAc–[EOA][OAc]) was developed for the efficient depolymerization of poplar polysaccharides, removing 88% hemicellulose and extracting around 46% lignin. An integrated OPP biorefinery model with California woody LCB, with [Ch][Lys] as a solvent, and *Saccharomyces cerevisiae* for sugar conversion promises a reduction in the ethanol price from $8.8 to $3/gasoline gallon equivalent (gge) [57]. The fermentation and saccharification conditions should have a similar pH, and [EOA][OAc] is an interesting IL because it can exclude pH adjustment [47]. Singh and collaborators used switchgrass and poplar as LCB, using OPP followed by hydrogenolysis. This process was found to be very promising for cellulose biofuel production in biorefinery schemes [58]. Konda et al. [59] checked in detail all the steps of two processes: 1. removing IL with water after treatment and 2. OPP, displaying a complete techno–economic analysis and concluding that the two processes are equivalent in terms of hydrolysis, but considering the use in the biorefinery, there is a tendency for OPP to be considered better because of the costs with water and sustainability of the process. 

## 5. Recovery, Reuse and Economic Feasibility of the Ionic Liquid

The recyclability of ILs is one of the great advantages that these solvents have. The reuse of ILs and the generation of considerable savings in the final process of biorefineries also make it environmentally sustainable [60]. The recovery of ILs is an alternative not only to their high cost, but also to their potential toxicity after pretreatment. The application of purification methods may be presented as an option for separating the degradation products absorbed into the liquor, allowing the recycling of the solvent. Several recovery methods have been investigated. There is no general ideal scope, as it is necessary to consider important variables, such as differences in the properties and composition of IL solutions and operational costs. Table 2 presents some works that recycled and reused ILs.

IL recovery and reuse are considered successful in consecutive extraction cycles when there is no loss of saccharification performance, there is a significant recovery, and biophysical analyses show that the IL structure is stable [70].

As seen above, laboratory studies demonstrate the efficiency of recycling and reusing ILs. In grass LCBs as well as eucalyptus, for example, the glucose yield was 75% in the first IL cycle and decreased to around 63% in the eighth cycle [37]. With rice straw, the initial performance was kept until the seventh IL reuse cycle, with just a 5% decrease in the eighth cycle [66]. Feasibly most critically, recycling processes and efficient separation will be required to cost-effectively recover ILs. A pervaporation system was used for 1-ethyl-3-methylimidazolium acetate dehydration, and separation was observed. The 99.9% of IL was recovered and reused five times, and the pervaporation membrane can be used over 60 dehydration cycles [71].

Understanding the cost drivers and economic potential of the variants of IL pretreatment for cellulosic biofuel production and the feasibility of reutilization of ILs in biorefinery processes still generate discussions. Economic studies have been carried out comparing processes that reuse ILs and OPP. Validation of these studies basically considers IL and recovery prices and LCB loading. Promising techniques have been demonstrated for high-throughput recovery and reuse of ILs from complex mixtures, including pervaporation, electrodialysis, and three-phase separation through the addition of salt solutions, but for a large scale, more studies are necessary [46]. 

Scale-up studies for the use of biomass dissolution by ILs should take into account several factors, such as the thermal stability of the IL, the separation of the main components of the LCB, recyclability, and operating costs [49].

One of the main obstacles to the use of ILs are the market prices practiced, which is why efficient pretreatment and recyclability protocols are essential [71,72]. Other operational challenges in scale-up must be taken into account: equipment can be resistant to corrosion, ILs can have high electrical conductivity, and this can be dangerous when in contact with wires, electrodes, or circuit boards [73]. For these problems to be avoided, the entire system must be chemical and heat resistant and with waterproof cables, for example Two chloride-based ILs were tested in a metal reactor in a recent scale-up study and showed elevated corrosion in the equipment [74].

Recent techno-economic analysis (TEA) of cellulose-dissolving IL pretreatment of LCB for fermentable sugars production includes pretreatment efficiency, recovery, and IL makeup costs, considering the key aspects to evaluate the viability of recovering the IL in a biomass pretreatment process. TEA highlights the importance of an integrated process evaluation to enable the design of cost-competitive biorefineries and is a model to evaluate and help improve the process, identifying challenges or bottlenecks. 

Ovejero-Pérez and collaborators performed an operational cost to IL recovery step in a real biorefinery pretreatment process as a function of the volume of water used in the pretreated *Eucaliptus globulus* LCB washing stage with two ILs: [Emim][OAc] and [Ch][OAc]. Better results for pretreatment efficiency, recovery, and IL makeup costs were observed when [Emim][OAc] was used. The work is sustainable if the IL is completely recovered [75]. Another interesting study was carried out using *Miscanthus giganteus* and the IL triethylammonium hydrogen sulfate [TEA][HSO_4_] for lignocellulose fractionation. Ninety-nine percent of the IL was recovered and reused four times, and the TEA predicted that the capital and operating cost was lower than for the reference dilute acid pretreatment [61]. A TEA to produce bioethanol and lignin applying the protic IL 2-hydroxyethylammonium acetate ([MEA][OAc]) showed that yield increased 33% and 5.6% to ethanol and lignin, respectively, when compared with other processes [76]. The TEA viability was studied on an integrated biorefinery for the co-production of furfural, lignin, and ethanol from switchgrass based on OPP. The ILs used for LCB treatment were aqueous choline chloride ([Ch][Cl]) and methyl isobutyl ketone (MIBK). Aspen Plus simulation indicated that 49% of the total carbon in the feedstock was converted to the target products (i.e., 17.9% to furfural, 16.0% to lignin, and 15.1% to ethanol). The proposed system indicates economic viability and commercial potential of a one-pot based system for biomass conversion into furfural and value-added co-products [77]. Sun et al. demonstrated a TEA to cellulosic ethanol from switchgrass in OPP, employing ethanolammonium acetate ([EOA][OAc]), a biocompatible IL in pretreatment. In this process, two steps were removed: the pH adjustment after the pretreatment and the water wash, and presented more than 40% of the minimum ethanol selling price (MESP) [47].

A few aspects, such as deep economic analysis, recycling, reuse, and reactivation use, still need to be addressed, discussed, and developed. The proper design of the reactors and establishing the scale-up rules for lignocellulosic pretreatment along with appropriate reaction kinetics and modelling of mass and heat transfers should also be studied. Biophysical, thermal, chemical, and structural properties studies during pretreatment of biomass should be promoted to successfully implement its large-scale application [4,47].

## 6. Final Remarks/Perspectives

ILs have been shown to be an excellent alternative as a green solvent for the pretreatment of LCB in the production of biofuels and other value-added products. This review shows different studies with very positive results related to the production of biofuels from LCBs, the importance of the reuse of ILs, and also OPP. The techno–economic analysis revealed that an integrated biorefinery concept based on one-pot and IL processes could potentially reduce the minimum ethanol selling price compared with scenarios that require pH adjustment prior to fermentation. Improvements in the economic performance will also be made by reducing the dilution, enzyme loading, and time of the operations, skipping the biomass washing step and avoiding solid–liquid separation between pretreatment and the hydrolysis step. Improving the technological route for the optimization of parameters in the pretreatment with ILs and the optimization of the OPP to obtain better results for the production of biofuels in shorter intervals and less costly processes are the challenges for the future.

## Figures and Tables

**Table 1:** Summary of the literature on LCB dissolution for biofuel production.

LCB	IL	Condition	Maximum Removal (%) ^a^	Glucose Yield (%) ^b^	Reference
Wheat straw	[Bmim][Cl]	100 °C, 5 h 130 °C, 2 h	28.3 Xylan; 9.9 Lignin 34.7 Xylan; 14.9 Lignin	37.3 37.8	[28]
Corn Stalk	[Bmim][BF_4_]	150 °C, 5 h	72.2 (30% IL) Xylan	81.7 (50% IL)	[29]
Sugarcane bagasse	[Bmim][OAc]	110 °C, 30 min (20:1)	22.5 Lignin 33.5 Xylan	96.5	[30]
Eucalyptus	[Bmim][OAc] + alkali treatment	120 °C, 30 min and 0.5, 2.0, and 4.0% NaOH at 90 °C for 2 h	17.0 Lignin 43.9 Glucan	90.5	[31]
Scots Pine	[Bmim][HSO_4_]/Water	170 °C, 4 h	64.0 Hemicellulose 55.0 Lignin	70.0	[32]
Yellow Pine	[Emim][OAc]	140 °C, 45 min	48.0 Glucan 30.0 Lignin	56.0	[33]
Radiata Pine	[Emim][OAc]	150 °C, 50 min	72.6 Hemicellulose	78.8	[34]
Softwood	[Emim][OAc]	100 °C, 60 min	25.6 Lignin 55.8 Glucan	34.0	[35]
Energy cane bagasse	[Emim][OAc]	120 °C, 30 min	32.0 Lignin	87.0	[28]
Energy cane bagasse	[Emim][OAc]	120 °C, 30 min	32.1 Lignin 43.9 Glucan 21.1 Xylan	68.0	[36]
Wheat straw	[Emim][OAc]	100 °C, 5 h 130 °C, 2 h	11.3 Xylan; 42.9 Lignin 58.9 Xylan; 50.6 Lignin	48.8 74.4	[37]

^a^ Maximum solubilization of the lignocellulosic fraction, according to the action mode of each pretreatment. ^b^ Glucose yield obtained from the solids pretreated after enzymatic saccharification.

**Table 2:** Recovery and recycling of ILs in biomass conversions.

IL	LCB	Recovery Method	Recycling Times	Saccharification Yield (%) ^a^	Ref
[Emim][OAc]	Cryptomeria japonica	Vacuum oven	3	46.2	[32]
[Ch][Arg]	Sugarcane bagasse	Evaporation	8	63.0–75.0	[37]
[Et3NH][HSO_4_]	*Miscanthus* × *giganteus*	Drying the IL solution	4	74.0	[61]
[Ch][phe]	Rice straw	Evaporation	5	70.2	[22]
[Amim][Cl] [Bmim][OAc]	Eucalyptus	Rotary evaporator/ Vacuum oven	4	54.3 72.8	[62]
[Emim][Cl]	Rice straw	Phase-separation process	5	86.0	[63]
[Ch][OAc]	Bagasse	Rotary evaporator	5	NS	[64]
[Emim][OAc]	Sugarcane bagasse		2	89.0	[65]
[Bmim][Cl]	Rice straw	Phase-separation process	8	98.9	[66]
[Bmim][OAc]	Pinus rigida	Vacuum drying	4	92.5	[67]
[Bmim][OAc]	Eucalyptus	Vacuum drying	4	72.8	[62]
[Emim][OAc]	Oak	Vacuum drying	8	53.7	[68]
[Emim][OAc]	Triticale	Lyophilization	2	81.0	[69]

NS: Data not shown in the work. ^a^ The reducing sugar yield of the sample pretreated with the last recycled ILs.

## Data Availability

Not applicable.
